# The Medical Department of the Michigan University and Our Cotemporaries

**Published:** 1855-05

**Authors:** 


*The Medical Department of the Michigan University and our Cotemporaries.*—From the peculiar position in which we are placed by recent official acts, we are constrained, though reluctantly, to bring before our readers again, matters we would be glad ever to sink in the presence of the deeply important truths of Medical Science.

Our readers will bear us witness that we have avoided allusions to ourselves and to the particular affairs of the Institution with which we are connected, excepting as impelled to it by evident necessity.

Until the issue of the March number we have had very little to say on the subject of Homœopathy, or any other of the various irregular systems of medical practice which are at the present day challenging public attention and soliciting public support. We have ever been of the opinion, as we still are, that these exclusive systems, like the long list which has superseded them, would, if left to their course, flourish their brief day and pass away—that their superstructure of errors and absurdities would be forgotton, and the grains of truth which each might chance to possess would be aggregated to the great mass of scientific knowledge.

Though avoiding to speak much of the Medical Department of the University, we have not been unconscious of the fact that the eyes of the Profession throughout the country were turned upon it,—some in envy, but most in hope. We have been aware that its position was new, at least in this country—that it was the only Medical School, on this side of the Atlantic, placed by the liberality of a State upon a basis independent of pecuniary patronage, and consequently free to pursue a course dictated alone by the wants and necessities of the community and the profession. We have been aware also of the uncertainty and instability of popular feeling—of the many influences which might be brought to bear upon a large body of men, such as the Legislature of a State, dependent for their position upon a variety of issues, and who, however pure in their motives and enlightened on general subjects, are still comparatively ignorant of Medical Science, of the feelings of the Medical Profession, and of the relations and necessities of a Medical School. In the multitude of Legislative acts, and the short time of forty days in which they are to be performed, it is not expected that the Legislature can give every subject a calm and full consideration, and matters of the nature of the late Act are entrusted to the care of committees, who will of course report in the absence of instructions, in accordance with their individual inclinations and views, whatever they may be.

The framers of the Constitution of our State, knowing the importance of an established system of management in regard to the University, and being aware of the state of things we have above referred to, placed the entire government of that institution in the hands of a separate body of men, more limited in number, elected for a long term of service, and having no other official duties to perform; and who it is supposed will make themselves familiar with all the relations of the subject of their charge, and of its necessities and wants. On the wisdom of this policy, and upon the good sense and honest purposes of the Board of Regents, we have rested in the greatest confidence, and we are happy to say that we have seen nothing to shake in the slightest degree that confident trust; and we hope after the present flurry has passed by, to be able to give our columns again entirely to the cause of Medical Science, and our time and thoughts in connection with the Medical College, to giving the best and fullest scientific and practical instruction to those under our care, of which we are capable. If we now step aside from our main object to give attention to these other matters forced upon us, our readers may be assured that as soon as possible, we shall again return to the calm and even tenor of our ways.

Some of our cotemporary journals, which have reached us, and whose editors had received intelligence of those recent official acts before their March issue, have noticed these occurrences, but in various tones.

The *Western Lancet* of Cincinnati, edited by a Professor in the Medical College of Ohio, has reached our table just at this moment, alluding to this subject, and to our journal in a most bitter and improper manner. While for our determined course in opposition to all exclusive systems, we are set upon by all the yelping pack of quackery let loose, this professional brother (!) most unjustly, as we think, accuses us of apologising for irregularities in Medical Science, and soundly berates us for advising our professional brethren to

"Seize on truth wherever found,
On foreign or on native ground."

We cannot say how much his feelings have been disturbed, by the small number of students in attendance upon the school with which he is connected, and how far this will account for the injustice of his accusations, but we fear he has a constitutional repugnance to the article which he abuses us for advising others to seize. We shall be obliged to regard this conclusion as settled, if, when the editor comes to understand our position, and read again the article he has condemned, he does not retract or modify the harsh statements he has made. We hope he will see that by some means he has been led into a mistake, and put himself right, as we would like to regard him, and all others laboring in the same cause, as gentlemen and honest men.

The *Buffalo Journal*, which hitherto has ever been ready to send a dart dipped in the bitterness which unsuccessful rivalry engenders, but whose darts have always been returned upon its own head by the impenetrable shield which surrounds us, until, to use its own confession, in was "just tottering to defeat," but by this act of the Legislature, and by a certain "report," possessing a sort of official character, concocted by men defeated in their hopes, and smarting under the loss of *honors* or powers and emoluments, either anticipated or previously possessed—the Journal by this act of the legislature, and this report, which, by the way, is harmless among those who understand the circumstances connected with its origin, has broken again its silence, and thinks it has found "confirmation strong as Holy Writ of our (its) prophecies of evil." It farther says:

"Admitting, as we do, most cordially, that the Medical Faculty of the Michigan University is mostly made up of men of zeal and attainment, amply qualified as teachers, it is nevertheless true that above these men are others, destitute of all attachment or veneration for medical science, who have the power to control the associations, if not the acts of the medical professors."

It inquires: "What are these professors to do? In the event of their resignation, what becomes of all their hopes of a great medical reform commencing at Ann Arbor? To resign is to give up a pleasant residence, a decent salary, and to abandon their cherished school to the control of Quacks. To remain is to submit to an insult degrading beyond measure, and to suffer under the consciousness that the next year the Eclectics will be entitled to professorship."

The Journal closes its article by saying: "We look upon the fulfillment of our predictions as a calamity which we ourselves would have labored strenuously to avert, and which subjects men whom we honor and respect to a most painful alternative."

It must be borne in mind that this article was written and printed before the March number of the *Peninsular Journal* reached Buffalo, and it reminds us of some obituary notices we have seen, written under erroneous telegraphic reports, and while the subjects of them were alive and kicking. Such notices, when written by opponents and rivals, often are in a strain which scarcely harmonizes with the tenor of previous effusions, but which show more truly the real sentiments and the manly heart. We really feel obliged to the editor of the Journal for his good opinions of the University and its professors, and would suggest to him that if he had always been as frank in his expressions as he has now, he would have avoided some of those "sore-headed rejoinders" of which he has so lively a remembrance, and which even still he seems to apprehend.

We have great pleasure in referring our new friend to the March number of the Peninsular Journal, where he will learn what we now more explicitly state, that "the men who are above us and have the power to control our associations, are *not* destitute of all attachment or veneration for medical science." We are happy to say, and we hope it will allay the regrets of the Buffalo and all other friends of the medical department of the University of Michigan, and of a "great medical reform," that the men who have been elected by the people of Michigan to control the University, have both an attachment to, and a veneration for medical science, and will not subject the men whom the editor of the Buffalo Journal so much honors and respects, to the "painful alternative he would have so strenuously labored to avert."

We are daily seeing new evidences of the profound wisdom of the great poet of human nature, and would suggest to Dr. Hunt that when he next quotes from Shakespeare he carefully consider the context. It will doubtless be worth his study. If in the present instance he had remembered the whole passage, and the desperate state of mind of Othello, of whom it was spoken, he would have found that,

> " *Trifles light as air,*
> Are to the *jealous,* confirmation strong
> As proof of holy writ;"

and he might have hesitated before giving so much consequence as he seems to have done, to the report of the chairman of the board of visitors, which we in Michigan understand better how to appreciate; and if his grief be not a luxury, might have saved himself a considerable amount of painful condolence.

One other of our cotemporaries deserves to be noticed, but the present article has already grown to such a length that we are unable at present to do it justice.—*Peninsular Journal of Medicine.*

